# Skin transcriptomics of invasive Coqui frogs: stress responses, parasite signatures, and antimicrobial peptides

**DOI:** 10.1371/journal.pone.0328623

**Published:** 2025-07-17

**Authors:** Randy Ortiz, Leeann C. Dabydeen, Carolyn Kosinski, Priyanka Gera, Juan D. Carvajal-Castro, Victoria Akilov, Hunter J. Howell, Emily Powell, Juan C. Santos

**Affiliations:** 1 Department of Biology, St. John’s University, Queens, New York, United States of America; 2 Department of Languages and Literatures, St. John’s University, Queens, New York, United States of America; 3 Department of Biology, University of Miami, Coral Gables, Florida, United States of America; University of Nevada, Reno, UNITED STATES OF AMERICA

## Abstract

Resilience in amphibians lies in their ecological adaptability, driven by their genetic makeup. *Eleutherodactylus coqui*, native to Puerto Rico (PR) and a beloved symbol there, is among the most successful invasive amphibians. This species is extensively studied in terms of its biology and genetics, including being the first *Eleutherodactylus* with a draft genome. Its potential to spread to new habitats and rapid breeding are notable. Transcriptome analyses of *E. coqui* are limited but provide insights into their invasiveness and differential gene expression. We compared the skin transcriptomes of *E. coqui* from PR (native) to those from an area under citric acid treatment in Los Angeles, California (invasive) population. Our results show differences in stress response gene signatures between both populations. In the native population, we hypothesize these responses are due to immunity against diverse parasites, potentially helping control their native populations in PR. Additionally, these coquis expressed several antimicrobial peptides, which were previously reported to be absent in coquis. These peptides may play a role in the invasiveness of the common coqui and its tolerance to urban and degraded habitats. We also provide novel draft transcriptomes of close relatives of *E. coqui*: *Eleutherodactylus planirostris*, *Eleutherodactylus johnstonei, Eleutherodactylus cochranae*, and *Pristimantis unistrigatus*.

## Introduction

Invasive species are a major factor in the decline of biodiversity around the globe, particularly in ecosystems already challenged by climate change and human activities such as deforestation, agriculture, and urbanization. Many amphibians suffer from these disturbances; however, some, like certain Caribbean frogs of the genus *Eleutherodactylus*, manage to thrive and become invasive. The most notable among these is *E. coqui*, known as the common coqui, a native, iconic, cultural, and cherished symbol of Puerto Rico (PR). This species is easily identified by its widespread presence and distinctive mating call “CO-KEE” in introduced areas. Due to their prolific nature, common coquis often reach high population densities, with a notable record of about 20,000 individuals per hectare at the El Verde field station in PR [1,2]. Such high densities are also observed in regions where common coquis have become invasive. Their chorus call is considered both a nuisance and an ecological threat, as they can outcompete or overconsume native invertebrates [3].

The history of the common coqui as an invasive species can be traced back to the unregulated trade of greenhouse plants from PR, primarily during the 1970s [4]. Since then, *E. coqui* has swiftly expanded beyond its natural habitat, initially as stowaways and later establishing populations in the southeastern US, CA, FL, TX, throughout the Caribbean, Hawaii (HI), and various Pacific islands [4]. Most introductions of the common coqui have been accidental, though there have been instances of deliberate release, such as in Costa Rica in 1998 [5]. Due to the common coqui’s pervasive presence, remarkable adaptability, reproductive capacity, and resilience, it has become the target of costly and radical eradication programs in areas where it is non-native. This intense focus has also spurred basic and applied research on the species, contrasting with the limited knowledge of the other ~200 members within the *Eleutherodactylidae* family. Studies on the common coqui encompass various aspects of its ecology, behavior, development, physiology, and taxonomy. Over the past 20 years, the species has also become a subject of molecular systematics, population genetics, and genomics research. To contribute to this effort, we aim to focus on the comparative transcriptomics of skin tissue, which serves as the primary barrier against pathogens and biopesticide agents used to control the spread of common coquis [6,7]. Our findings could provide insights into the differences between natural and invasive populations of *E. coqui*, and how genomic and transcriptomic tools can illuminate the species’ resilience against pathogens and its invasive potential.

Over the past decade, next-generation sequencing technologies have significantly enhanced our understanding of the molecular basis of invasiveness in plants and animals [8]. In the case of the common coqui, most research has centered on skin swabs, which have been instrumental in exploring various aspects of the species’ health, microbiome, skin excretions, and skin cell content. For example, proteomic studies have not detected the presence of antimicrobial peptides (AMPs), leading to the conclusion that coquis might lack these defenses [9]. In studies on native *E. coqui*, skin samples were collected and analyzed using matrix-assisted laser desorption/ionization time-of-flight (MALDI-TOF) mass spectrometry [9]. The researchers, using skin swab samples, were unable to detect any AMPs and concluded that the common coqui might lack these powerful innate defenses against a variety of pathogens, including the frog pandemic agent *Batrachochytrium dendrobatidis* (also known as Bd or chytrid fungus) [9]. However, they noted that since *E. coqui* is resilient against Bd, other mechanisms might be at play.

The most notable genomic work on *E. coqui* is a public draft genome that spans all 13 chromosomes, totaling 2789.35 Mb (NCBI UCB_Ecoq_1.0 accession number GCA_019857665.1). This genome was obtained from an invasive adult male (HN-11) collected in Hawaii, using kidney and liver tissues [10]. The chromosomes of *E. coqui* were found to be highly conserved compared to other frog genomes, including *Engystomops pustulosus*, *Hymenochirus boettgeri*, *Leptobrachium ailaonicum*, *Pyxicephalus adspersus*, *Xenopus laevis*, and *X. tropicalis* [10]. Like most first drafts, the *E. coqui* genome has 8% missing nucleotide sequences (represented as Ns), amounting to over 230 Mb. Some of these sequences might be found in the 105,220 unplaced scaffolds, which account for approximately 1007 Mb. Despite these limitations, the *E. coqui* genome exhibited signs of sequence divergence.

While a genome reveals the identity and sequence structure of an organism’s genes, it offers limited insight into transcript variability, alternative gene splicing, and tissue-specific expression [11]. Conversely, transcriptomes provide such genetic profile data, offering molecular answers to ecological questions. However, transcriptomes represent a snapshot of a specific moment in an organism’s life, meaning not all genes are always expressed [11]. Additionally, transcriptomes include data on ncRNA sequences and transcript isoforms. A recent study by Laslo [12] presented the first whole-body transcriptome of *E. coqui*, focusing on the species’ endocrine-mediated development. This work highlighted *E. coqui* as an excellent model for studying developmental biology (direct development), neuroethology (mating behavior), and invasion biology, given the extensive research at the species and population levels [13]. However, to our knowledge, no current studies provide a comprehensive transcriptome assembly for skin or compare invasive and native populations, nor do they explore which genes might contribute to the coqui’s resilience, such as AMPs or other immunity mechanisms [12].

Our study provides a comparative transcriptome analysis of total skin from native *E. coqui* in Puerto Rico (PR) and an invasive population in an area under citric acid treatment in Los Angeles, California (LA-CA). By examining transcriptomic data from both populations, we aim to identify genetic signatures of parasites that could influence population control if present; their absence, however, may contribute to enhanced reproduction and dispersal [14]. Additionally, we investigate whether *E. coqui* truly lack AMPs as suggested in the literature and assess indicators of acute metabolic stress.

## Methods

### Sample collection and transcriptomic sequencing

We collected three individuals from an invasive *Eleutherodactylus coqui* (*E. coqui*) population in Torrance, California, within the Los Angeles metropolitan area (Los Angeles County, California, United States). This area had previously been exposed to citric acid spray, the standard biopesticide used to eradicate invasive coquis in the US [7]. Additionally, we collected four native *E. coqui* from the Río Blanco locality in Naguabo, Puerto Rico (PR, 18.261189, −65.796228) to serve as our reference population. For comparison, we also included five individuals from two invasive populations of other *Eleutherodactylus* and *Pristimantis* species: four individuals of *Eleutherodactylus johnstonei* (*E. johnstonei*) from Cali, Colombia, and two individuals of *Eleutherodactylus planirostris* (*E. planirostris*) from Miami-Dade, Florida (FL). We also analyzed individuals from non-invasive related species, including one individual of *Eleutherodactylus cochranae* (*E. cochranae*) collected from Río Blanco, PR, found in sympatry with our native *E. coqui* population, and three individuals of *Pristimantis unistrigatus* (*P. unistrigatus*) from its native distribution in Quito, Ecuador.

All collected individuals were kept in containers with leaves and processed within 2–4 hours. We followed the humane euthanizing methods [60,61] described in the IACUC protocol IACUC-SJU # 1965 and approved by St. John’s University IACUC committee 2020 to JSC and laboratory members. Anurans were humanely sacrificed through pithing and subsequent severance of the spinal cord. Tissue collections included skin and internal organs, which were preserved in RNAlater at 4ºC for the first 24 hours. For long-term storage, the samples were kept in a −20ºC freezer. Collection permits were issued by Puerto Rican authorities: DRNA#: 2021-IC-011, O-VS-PVS15-SJ-01139–22072020.

Each specimen was processed separately. Total RNA was extracted from skin tissue preserved in RNAlater using the Trizol RNA extraction protocol after tissue liquification with triple-pure zirconium beads [15]. The resulting total RNA extractions were quantified using a Nanodrop and stored at −80ºC until submission for sequencing. After assessing integrity with a bioanalyzer, only samples with a RIN > 7.0 were used for further mRNA isolation, followed by directional RNA-seq library preparation, and Illumina 150 bp paired-end sequencing. Briefly, mRNA was isolated using oligo(dT) beads, and rRNA was removed using the Ribo-Zero kit. The isolated mRNA was then used for cDNA synthesis and 150 bp paired-end sequencing, achieving total coverage of 20–30 million sequence reads. The raw sequence data used for our further analyses were submitted to the NCBI-SRA database under the BioProject PRJNA953648.

### Skin transcriptome reconstruction, annotation, and expression

Raw RNA sequence reads (Table 1) were processed using Pincho v0.1 [16]. This software offers a bioinformatics pipeline for high-quality transcriptomics, automating the following steps: (1) Removal of Illumina adapter sequences with Trimmomatic v0.39 [17]; (2) Insert error correction with Rcorrector v1.0.4 [18]; (3) Three-assembler reconstructions using trans-ABySS v2.0.1 [19] and rnaSPAdes v3.14.1 [20] with five computationally generated k-mer sizes derived from adapter-clean data inserts, and TransLig v1.3 [21]; (4) Combined consensus transcriptome assembly derived from the three-assembler reconstructions using TransRate v1.0.3 [22]; (5) Identical transcript redundancy reduction with CD-HIT v4.8.1 [23], and consensus assembly quality assessment with BUSCO v4.0.1 [24] for the eukaryota_odb10.2019-11-20 dataset; and (6) Transcript annotation against TrEMBL: amphibia with BLASTx v2.10.0+ [25], annotation against UniProt: Swiss-Prot with BLASTx with an e-value of 1e-10, and annotation against KEGG: *Xenopus laevis* and *Nanorana parkeri* with BLASTn with an e-value of 1e-10. For each transcript with a database match, only the first reference with the longest sequence and the lowest e-value was used for annotation.

**Table 1:** Illumina NGS skin sequence data and assembly metrics [26].

Submission ID	Organism	Collection Location	Number of Reads (in millions)	Insert Length (bp)	Number of Bases (Gbp)	Busco Completeness After Assembly
CC22	Invasive *E.coqui*	Los Angeles California	25.1	150	3.8	98.1%
CC41	Invasive *E.coqui*	Los Angeles California	25.2	150	3.8	96.5%
CC52	Invasive *E.coqui*	Los Angeles California	22.2	150	3.3	97.7%
PC1−1	Native *E.coqui*	Naguabo Puerto Rico	41.2	150	6.2	98.1%
PC3−1	Native *E.coqui*	Naguabo Puerto Rico	32.5	150	4.9	99.2%
PC5−1	Native *E.coqui*	Naguabo Puerto Rico	25.4	150	3.8	97.3%
PC8−1	Native *E.coqui*	Naguabo Puerto Rico	31.2	150	4.7	98.1%
CJ1	Invasive *E. johnstonei*	Cali Colombia	58	150	8.6	83.5%
CJ2	Invasive *E. johnstonei*	Cali Colombia	35.3	150	5.2	97.3%
CJ3	Invasive *E. johnstonei*	Cali Colombia	39	150	5.8	89.8%
CJ4	Invasive *E. johnstonei*	Cali Colombia	33.1	150	4.9	98.4%
CP1.1	Invasive *E. planirostris*	Miami Florida	49.2	150	7.3	97.7%
CP1.2	Invasive *E. planirostris*	Miami Florida	14.2	150	2.1	94.1%
CP2.1	Invasive *E. planirostris*	Miami Florida	23.3	150	3.4	94.1%
CP2.2	Invasive *E. planirostris*	Miami Florida	14.4	150	2.1	91.7%
PU1	Native *P. unistrigatus*	Quito Ecuador	28.8	150	4.3	96.5%
PU2	Native *P. unistrigatus*	Quito Ecuador	29.5	150	4.4	98.1%
PU3	Native *P. unistrigatus*	Quito Ecuador	35.1	150	5.2	98.8%
EC1.1	Native *E. cochranae*	Naguabo Puerto Rico	27.4	150	4.1	97.7%
EC1.2	Native *E. cochranae*	Naguabo Puerto Rico	28.6	150	4.2	97.7%
EC1.3	Native *E. cochranae*	Naguabo Puerto Rico	32.5	150	4.8	98.4%

To enhance our transcriptome annotation for differential expression analysis, we included an additional re-annotation step using a reference library limited to *Xenopus tropicalis* (characterized UniProt) on the consensus assemblies with DIAMOND BLAST v2.0.11 [27]. This step allowed us to isolate single gene targets and trim reconstructed transcripts to match only the *Xenopus tropicalis* database entries. Input transcripts are aligned via DIAMOND BLAST to the reference library and only the aligned region is retained, the unaligned regions are removed. This alignment-based approach effectively removes chimeric gene fragments, as only the aligned region remains, ensuring accurate transcripts for subsequent expression analysis. Duplicate transcripts were removed using BBMap’s dedupe protocol v38.86 [28], which eliminated partial reconstructions if a complete or longer matching sequence was present in the transcriptome database.

We compared three invasive coqui samples against four native coqui samples, and each of these organisms against invasive *E. johnstonei*. For gene expression analyses, weighted transcript/contig abundance was determined using the annotated transcriptome with Salmon v1.5.2 [29] with default parameters. Data tables from these counts were used for expression analyses with DESeq2 [30] in the DEBrowser webtool [31] to visualize our results as a volcano plot with default settings of 5% false discovery rate and p value of 0.05. Read counts below ten were filtered out before analysis to reduce noise due to sequencing errors or low-abundance transcripts. We then used Panther17.0 [32] with the reference organism of *Xenopus tropicalis* to identify significant gene ontology groups, using protein class option and our annotated entry IDs, from the over- and under-expressed gene results from DESeq2.

### Parasite detection and mitogenome

For parasite detection, we focused on *E. coqui* as we had both the native and invasive populations along with expression analysis data. For our parasite database, we downloaded a list of UniProt IDs for nematodes and platyhelminths. Using this list, we filtered out parasite genes from our *E. coqui* transcriptome UniProt: Swiss-Prot annotations. Matching transcripts were further refined to mitochondrial proteins, which were then validated via BLASTn against the complete NCBI nucleotide database at an e-value of 1e-10, yielding 18 distinct sequences (S1 Table).

We also reconstructed the mitogenomes from RNAseq data, which are common by-products of transcriptome assemblies. To isolate these genomes, we filtered the annotated transcripts from the UniProt: Swiss-Prot consensus transcriptome assemblies of *E. coqui*, *E. planirostris*, *P. unistrigatus*, and *E. cochranae* (S1 Fig). If a mitogenome was already reconstructed in our UniProt: Swiss-Prot consensus skin transcriptome (e.g., PR coqui, validated against *Eleutherodactylus atkinsi* JX564864.1 reference partial mitogenome), we further validated this mitogenome using the ‘annotate’ command of MitoZ software v3.4 [33] with the raw Illumina reads for the corresponding species.

For our target *Eleutherodactylus* and *Pristimantis* species, their annotated transcriptomes contained sequences matching a mitogenome to near complete length (approximately 17 kb). The best reconstruction was used as a seed for further validation with the MitoZ assembler under default parameters. MitoZ provided an output file with a GenBank-ready submission file, including all tRNA, rRNA, and mitochondrial protein-coding genes delimitations; a stack-map of clean sequence reads that match the target/seed mt-genome; and a circular plot of each mt-genome of the target species.

If no complete mitogenomes were reconstructed using MitoZ, we explored the corresponding unannotated transcriptomes using MMseqs2 [34] to retrieve the best and longest mitogenome transcript after comparison with the reconstructed *E. coqui* and *E. atkinsi* mitogenomes as references. This step allowed us to further validate partial mitogenome MitoZ reconstructions and create a consensus mitogenome for our target *Eleutherodactylus* and *Pristimantis* species when no single method reconstructed the full mitogenome.

## Results

### Draft skin transcriptomes

All seven *E. coqui* skin transcriptomes and four *E. johnstonei* skin transcriptomes were assembled and achieved BUSCO scores greater than 96% (with the exception of two *E. johnstonei* transcriptomes that scored between 80–90%), indicating high overall transcriptome completeness (Table 1). Using a combination of sequence references (UniProt: *Xenopus tropicalis*, UniProt: Swiss-Prot, TrEMBL: Amphibia, and KEGG: *Xenopus laevis* and *Nanorana parkeri*), we identified 62,512 and 76,241 unique transcripts (including unique genes and isoforms) in the main target organisms of interest LA-CA *E. coqui* and native *E. coqui*, respectively (Table 2). When combining all samples, we collectively found a total of 81,859 unique transcripts between the two populations. Duplicate sequences were removed from the final annotations, although duplicate gene IDs were not excluded. The UniProt: *Xenopus tropicalis* annotation was trimmed to match coding sequences (CDS) to separate chimeric/fused genes. Transcriptomes for *E. planirostris*, *E. cochranae*, and *P. unistrigatus* were also assembled with the same methodology, scoring >90% BUSCO scores for transcriptome completeness.

**Table 2:** Native and invasive *Eleutherodactylus coqui* unique annotated alleles [26].

Collection	Uniprot: *Xenopus Tropicalis*	Uniprot: Swiss-Prot	Trembl: Amphibia	Kegg: *Xenopus Laevis* and *Nanorana Parkeri*
California *E. Coqui*	17,723	22,634	37,675	12,183
Puerto Rico *E. Coqui*	18,144	26,937	50,949	16,695

**Fig 1:**
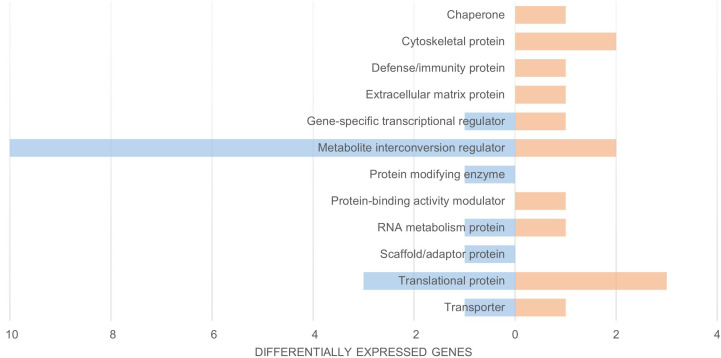
Functional Classification via Protein Class of Invasive and Native *E. coqui* [26]. Panther17.0 function classification of DESEQ2 expression data. Transcript count number for each protein class denoted on the x-axis. Native *E. coqui* are represented in orange and invasive *E. coqui* are represented in blue.

To further explore the differences between coqui populations, we utilized Panther17.0 protein class ontology of each differentially expressed transcript between invasive and native coqui samples to create a general functional classification for over- and under-expressed transcripts (Fig 1). In our comparisons, we identified several functional groups that were overexpressed in native coqui but not in the invasive population. These gene groups include chaperone, cytoskeletal, defense/immunity, extracellular matrix, and protein-binding activity modulator proteins, which are typically indicative of an active response to infection or parasitism [35,36]. The overexpressed immune response in native coqui suggests that they may harbor parasites or be actively responding to such infections.

In contrast, in the invasive population, we observed overexpression of genes related to modifying enzyme and scaffold/adaptor processes, when compared to the native population. The invasive coqui individuals were collected from an area recently sprayed with citric acid, so we anticipated the over-expression of stress-related genes in this group. Both populations, when compared to each other, exhibited overexpression of different sets of stress-related genes, such as transcriptional regulators, metabolite interconversion regulators, RNA metabolism proteins, translational proteins, and transporter proteins. However, only the invasive coqui showed a significant overexpression of metabolite interconversion regulators compared to native coqui. Among these metabolite interconversion regulator proteins, we identified several related to metabolic stress, including seven oxidoreductases and three transferases, indicating an increased metabolic rate possibly due to contact with citric acid.

**Fig 2:**
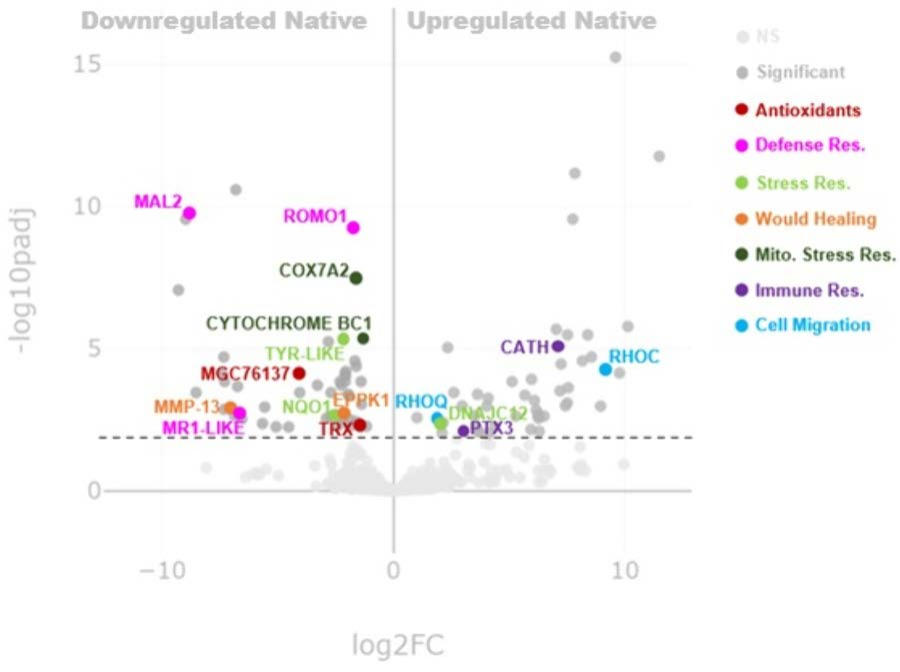
Differential Expression (DE) Analysis Comparing Native to Invasive *E. coqui* [26]. Nonsignificant transcripts under p < 0.05 are depicted in light gray. Significant (p > 0.5) transcripts are depicted either in dark gray or in non-light gray color. Transcripts of interest and the gene ontology groups of antioxidants (red), defense response (defense res.; pink), stress response (defense res.; light green), would healing (orange), mitochondrial stress response (mito. stress res.; dark green), immune response (immunes res.; purple) and cell migration (light blue) are labeled, and color coded accordingly. DE was conducted via DESeq2 with default parameters. Upregulated transcripts in native coqui are on the right, while downregulated transcripts are on the left. Low read counts under ten were filtered prior to analysis. 102 total DE genes are depicted.

Using the coqui CDS aligned with the UniProt: *Xenopus tropicalis* (*X. tropicalis*) annotation, we conducted an expression analysis by contrasting the LA-CA versus PR populations. Using a complete genome from a model organism provides a well-defined reference framework, including precise gene boundaries, exon-intron structures, and robust functional annotations,which enhances the accuracy of gene alignment and gene quantification. This is a unique advantage as a de novo transcriptome can contain fragmented or partially assembled transcripts, leading to ambiguous alignments and incomplete annotations. As a result, referencing a curated genome often allows for the identification of additional genes that may be missed in de novo assemblies and improves confidence in differentially expressed gene (DEG) calls. This approach enabled us to further refine the differentially expressed genes and even identify others that were not annotated using the standard UniProt: Swiss-Prot reference database. By using native coqui from PR as a reference, we found 102 differentially expressed genes, with 49 genes under-expressed and 53 over-expressed in the PR population compared to the invasive LA-CA population (Fig 2 and S2 Table). It is also important to note that this approach may limit the detection of novel species-specific transcripts that are not conserved between *X. tropicalis* and *E. coqui*, due to their large phylogenetic distance from each other, which would be captured via de novo transcriptome assembly. Thus, only conserved genes between the two organisms would be reported in our expression analysis data.

Among these, Panther17.0 predicted three significant transcripts (A0A6I8Q9X7, A4QND9, and Q6PBF4) from the invasive population, classified as mitochondrial ATP synthesis coupled electron transport related proteins. However, our Panther17.0 analyses could not identify any significant grouping for biological processes in the native population. Overall, these results suggest that the LA-CA individuals were more metabolically active, yet most differences between LA-CA and PR were not apparent at the gene expression level.

**Fig 3:**
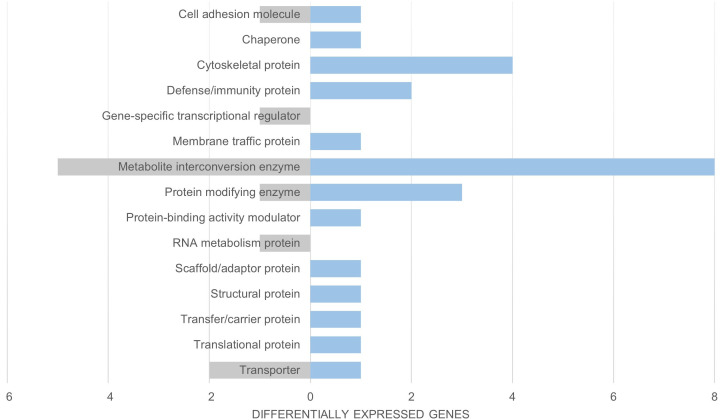
Functional Classification via Protein Class of Invasive *E. johnstonei* and Invasive *E. coqui* [26]. Panther17.0 function classification of DESEQ2 expression data. Transcript count number for each protein class denoted on the x-axis. Invasive *E. johnstonei* are represented in gray and invasive *E. coqui* are represented in blue.

**Fig 4:**
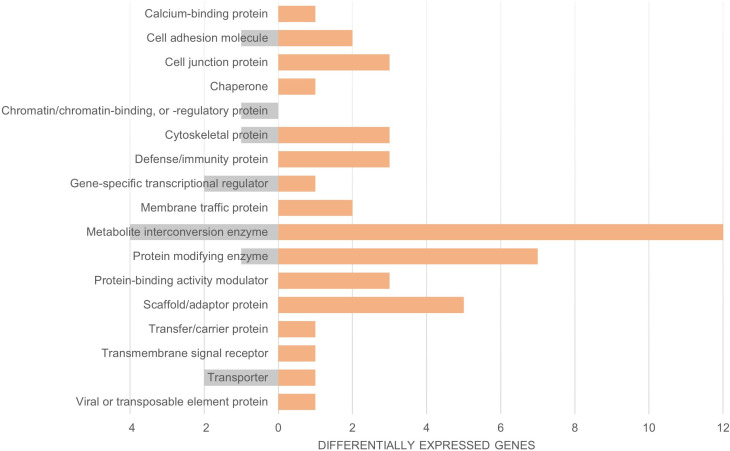
Functional Classification via Protein Class of Invasive *E. johnstonei* and Native *E. coqui* [26]. Panther17.0 function classification of DESEQ2 expression data. Transcript count number for each protein class denoted on the x-axis. Invasive *E. johnstonei* are represented in gray and native *E. coqui* are represented in orange.

We compared both the invasive and native common coqui skin transcriptomes with that of *E. johnstonei* and our results did not show overexpression of defense/immunity peptides in invasive *E. johnstonei* compared to our samples of native or invasive coqui (Figs 3 and 4). This may reflect the fact that *E. johnstonei* were not exposed to citric acid or any other form of biopesticide control and were not parasitized in their introduced Colombian location. Consequently, *E. johnstonei* individuals were not under stress or combating infections when collected for transcriptome profiling.

### Antiparisite defenses and stress responses

**Fig 5:**
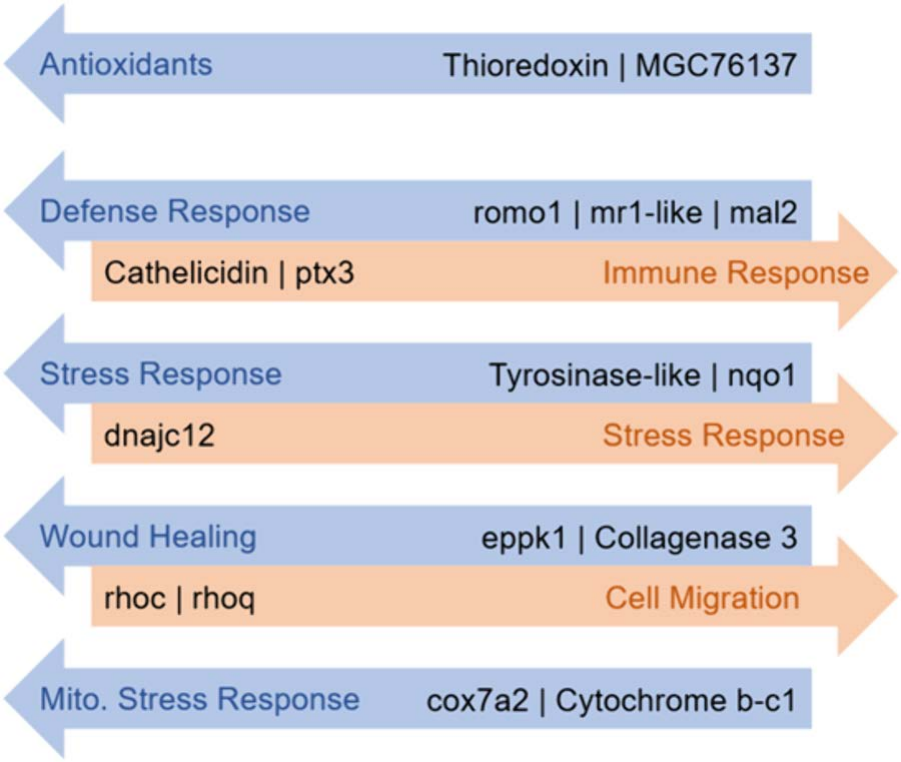
Genes of Interest in Native *E. coqui* [26]. DESEQ2 expression analysis results between native *E. coqui* and invasive *E. coqui*. Overexpressed genes in the native population are represented in orange. Unexpressed genes in the native population are represented in blue.

Since Panther17.0 relies on a GO-Slim subset, which can omit more detailed annotations, we revisited the differential gene expression results between native and invasive coqui to identify gene ontology groupings that were left unreported. Among all differentially expressed (DE) genes, we identified cathelicidin (CATH) and pentraxin 3 (PTX3) as two key immune response genes (Fig 5). Both CATH and PTX3 were overexpressed in PR coqui, with CATH being entirely absent in the invasive LA-CA coqui transcriptomes (Fig 5 and S2 Table). Additionally, reactive oxygen species modulator 1 (ROMO1), major histocompatibility complex class I-related-like (MR1-LIKE), and mal-T cell differentiation protein 2 (MAL2) were identified as general defense response genes (Fig 5). ROMO1, MR1-LIKE, and MAL2 were overexpressed in LA-CA coqui, with MR1-LIKE and MAL2 completely absent in PR coqui (Fig 5 and S2 Table).

The native coqui from PR showed evidence of stress responses, including overexpression of the protein DNAJ heat shock protein family HSP40 member C12 (DNAJC12), cell migration genes RAS homolog family member C (RHOC) and RAS homolog family member Q (RHOQ), and oxidative phosphorylation mitigation genes COX7A2 and CYTOCHROME B-C1 when compared to the invasive population (Fig 5 and S2 Table). In contrast, the invasive LA-CA coqui seemingly exhibited a response to citric acid spray, evidenced by the overexpression of antioxidant peptides such as thioredoxin (TRX), MGC76137, and peroxiredoxin 6 (PRDX6). These individuals also showed signs of skin damage, indicated by the increased expression of wound healing genes such as epiplakin 1 (EPPK1) and collagenase 3 (MMP-13), and stress mitigators tyrosinase-like (TYR-LIKE) and NADPH dehydrogenase quinone 1 (NQO1; Fig 5). Such wound healing responses were not observed in the native coqui from PR, as shown by the limited or absent expression of MMP-13 (S2 Table). In response to the observed infection in native coqui, we identified overexpressed immunity-related genes in both populations. Notably, DMBT1, FAM3A, and CATH, transcripts were detected exclusively in the native coqui population, while none were found in the invasive population.

### Nematode signatures in *E. coqui* transcriptomes

We identified a diverse range of parasite/commensal invertebrate species in our UniProt annotation of native *E. coqui* from Puerto Rico. This includes 16 sequences matching nematode mitogenomes. However, even the sequence with the best BLAST max score and the lowest e-value of 0 matched at least 60 distinct species. Therefore, we present 10 distinct species based on the BLAST max score, acknowledging that these may not be the exact parasites within the PR coqui. These species include the following roundworms: *Ascaris suum*, *Anisakis simplex*, *Angiostrongylus cantonensis*, *Strongylus equinus*, *Oesophagostomum quadrispinulatum*, *Toxocara vitulorum*, *Oscheius tipulae*, *Pseudoterranova azarasi*, *Contracaecum osculatum B* sensu Nascetti et al. (1993), and *Contracaecum* sp. ALS-2019 (S1 Table). We present this biodiversity knowledge as evidence of distinct parasite/commensal invertebrate signatures within PR coqui, with no similar molecular signatures detected in the invasive CA coqui.

### Mitogenomics

We reconstructed a near-complete *E. coqui* draft mitogenome containing 35 out of 37 total genes. The only missing genes were *trnL* and *trnF*, which encode tRNAs (S1 Fig). These tRNAs might be present in mitogenome sequences that did not align well with the *Eleutherodactylus* reference. We successfully recovered cytochrome c oxidase subunit 1 (COI) transcripts from all three samples of LA-CA coqui, and these transcripts were identical to each other. All three COI sequences from LA-CA coqui mapped best to an *E. coqui* voucher for COI (KY033486) collected from Kukuihaele, Hawaii, with 98.72% sequence identity and 100% query cover, providing strong evidence of their relatedness.

## Discussion

We studied the transcriptomes of *E. coqui* individuals from both native (Puerto Rico) and invasive (Los Angeles, CA) locations. This approach enabled us to perform differential expression analyses and identify genes relevant to stress, immunity, defenses, and even signatures of parasitic infestation. Additionally, we reconstructed the mtDNA genome of these *E. coqui* to determine the likely origin of the invasive population in Los Angeles, CA, and its relationship with other native and invasive populations. Given the breadth of our results, we emphasize that transcriptomic data can provide a comprehensive overview of the biology and invasive potential of any species with a well-documented life history.

Individuals of the invasive LA-CA population of the common coqui, collected from an area under citric acid treatment, exhibited upregulation of wound healing genes in transcriptome analyses, suggesting a possible response to partial contact with citric acid, though other factors could also explain this response and further studies are needed to confirm the association. These California coquis exhibited high levels of wound healing genes, such as EPPK1 and MMP-13, along with stress mitigation genes like antioxidants TRX and PRDX6, which were not overexpressed in the PR native population of coqui. EPPK1 assists in keratinocyte migration during wound healing, and MMP-13 breaks down extracellular matrix proteins for tissue remodeling. TRX reduces disulfide bonds in proteins, and PRDX6 reduces hydrogen peroxide and organic hydroperoxides, both protecting cells against oxidative stress.

The cause of the wound healing response is unclear; however, the history of the area where we collected the samples provides some clarity, though it may be coincidental. At the time of collection and even currently, 16% citric acid treatments are used in California as a minimum-risk pesticide to euthanize coqui [7]. This pesticide eliminates coqui at any life stage upon direct contact with 100% effectiveness [7]. Citric acid is quickly absorbed by coqui skin, sending the frog into osmotic shock within 30–60 minutes; however, partial or indirect contact may not be lethal [7].

The invasive coqui from California were collected from an area in Torrance undergoing citric acid treatment. These coquis were kept overnight, indicating they were not killed by the citric acid treatment; however, we cannot rule out that they were not indirectly or partially exposed to the pesticide. Regardless, our results support that citric acid may induce metabolic stress on the common coqui, as the overexpression of wound healing and antioxidant genes likely represents a response to cellular damage caused by the acidity and toxicity of the citric acid solution.

Upon further inspection utilizing *E. johnstonei* as a third group in our differential expression analysis against native and invasive *E. coqui*, we noted several key takeaways. The sampled *E. johnstonei* population, although invasive, does not show overexpression of any wound healing peptides. Since this population was not under citric acid treatment nor did we observe any noticeable wounds, this further supports our hypothesis regarding citric acid treatment and the wound healing response. Additionally, we observed a lack of overexpression of immune peptides in *E. johnstonei*, indicating that these anurans are not currently combating infections as our native *E. coqui* were. Upon closer examination of the *E. johnstonei* transcriptome, we were unable to identify any actively expressed cathelicidin sequences, unlike in *E. coqui*, *E. cochranae*, and *E. planirostris*. This absence further highlights the unique responses and adaptations of *E. coqui* and its relatives under different environmental pressures and treatments.

Our analysis of the native coqui transcriptomes revealed several unknown species of roundworm. Many of these taxa are likely endoparasites, as several mitochondrial transcripts were similar to those found in causal agents of serious illnesses in aquatic and terrestrial animals. Notably, *Angiostrongylus cantonensis* roundworms have been found previously in other *E. coqui* from Hawaii, as well as in other anurans [37,38]. These parasitic nematodes are known to be transmissible to humans via intentional or unintentional ingestion. For instance, there was a reported case in Thailand where human consumption of an infected frog led to the onset of eosinophilic meningitis [37,39]. Other parasitic nematodes identified in the transcriptome of *E. coqui* included sequences similar to *Anisakis simplex*, which causes anisakiasis. These roundworms typically infect fish and marine mammals but can also infect humans. We detected *Anisakis* in native coqui, and it has also been found in wild-caught marine fish and *Rana esculenta* [40]. Another parasitic worm identified was *Ascaris sp.*, which, found in *Rana areolate*, can cause malnutrition, developmental issues, and, rarely, death in humans [41,42]. Additionally, we found *Contracaecum sp.*, which can cause anisakidosis in humans, although members of this genus tend to be specific to amphibians [43,44]. Other sequences matched *Oesophagostomum quadrispinulatum*, which can parasitize humans [45]; *Strongylus equinus* (also known as *Strongylus vulgaris*), found in frogs, which can rarely cause verminous encephalitis in horses if it migrates to the brain or thrombosis of the smaller arteries around the brain [46]; and *Toxocara vitulorum*, linked to toxocariasis in humans [47].

Utilizing *E. coqui* skin samples from Puerto Rico and LA-CA, we identified several overexpressed genes in both populations that indicate high levels of stress mitigation. In PR coqui, we observed high levels of immune response and general stress response genes, most notably a significant presence of CATH, a potent AMP. In contrast, LA-CA *E. coqui* exhibited no expression of CATH or any other AMP. These peptides are known to act directly on microorganisms and indirectly as cell-signaling molecules [48]. Previous research had been unsuccessful in identifying any AMPs within *E. coqui*, suggesting that coquis rely on other mechanisms for pathogen protection; however, many CATH sequences are found in the *E. coqui* genome [9].

CATH are known to defend skin tissue against a wide range of bacteria, viruses, and fungi, as they are highly expressed in epithelial cells, neutrophils, dendritic cells, lymphocytes, mast cells, macrophages, monocytes, and NK cells in vertebrates [48–53]. CATH are also produced in dermal serous glands, where they are stored within granules until released [54]. They can exert direct antimicrobial effects and trigger specific defense responses within the host [55]. The cationic and amphipathic properties of CATH allow them to associate with the negatively charged phospholipids in bacterial membranes, penetrate the invading microbe’s membrane, leading to fragmentation of the membrane and subsequent cell death [48,49].

The CATH gene in *Rana muscosa* and *Rana sierrae* was found to be one of two AMPs overexpressed in response to Bd infection [56]. We believe that this newfound CATH may also play a significant role in *E. coqui*’s resilience to Bd infection, as *E. coqui* is resilient against Bd, although we did not detect the presence of Bd infection in our samples [9]. Instead, we detected the mitochondrial signature of ten roundworms within PR coqui, any of which may have triggered CATH production.

It has been noted that CATH produced by amphibians are species-specific and vary tremendously between species [57]. CATH is generally grouped into five major types based on amino acid composition: glycine and serine-rich or type-GS, proline and arginine-rich or type-pr, tryptophan and arginine-rich or type-wr, β-haripin or type-β, and α-helix or type-α [50]. Anuran CATH active peptide regions are typically type-GS; however, *E. coqui* CATH contains a glycine-arginine-rich highly repetitive region (NGGRG) in its active peptide region that breaks from this classification. This type variability leads us to believe *E. coqui* CATH may be different than previously described CATH peptides.. This is of great interest considering CATH’s potential applications in wound healing, angiogenesis, antimicrobial, anti-inflammatory, and anti-cancer research [57]. We propose exploring these unique CATH peptides is a great area for further research.

We also noted a significantly higher level of PTX3 in the native coqui population compared to the invasive population. Long pentraxin PTX3 is produced by endothelial cells and macrophages [58]. PTX3 plays a role in innate immunity by binding to both gram-negative bacteria and fungal spores in mouse models [59]. The significantly higher levels of PTX3 in the native population support our hypothesis that the native coqui is in an environment more saturated with pathogens compared to the environment where they are invasive.

Finding high amounts of CATH and PTX3 suggests that the environment in Puerto Rico (PR) may not be as ideal for *E. coqui* as the locations where they have become invasive and expansive in which parasites may be scarcer (e.g., Hawaii, where the highest population densities are observed) [14]. Managing environmental stressors, including but not limited to infections, may be one of several factors limiting the population density of *E. coqui* in PR compared to California and Hawaii. This might also explain the underexpression of several oxidoreductases in the PR population, indicating decreased metabolic activity [60]. It is likely that CATH and PTX3 played roles in defending the organism by directly interacting with stress factors or by signaling local cells to initiate protective responses.

Our study provides the first skin draft transcriptome assembly of *E. coqui*, a non-model organism, revealing molecular processes and making these transcriptomes publicly available to support research on its resilience to infections like Bd, invasiveness control, and conservation in native Puerto Rico (PR) and introduced regions like California (CA). Differential expression analysis suggests *E. coqui* employs a novel form of antimicrobial peptide (CATH) and immune mediator (PTX3) as primary defense mechanisms against pathogens entering the skin. Comparing native and invasive populations, we found higher stress levels and a greater diversity of roundworm infections in PR coqui, absent in CA coqui, indicating suboptimal local conditions. However, invasive coqui escape pathogen-rich environments, aligning with the “enemy release hypothesis,” which hypothesizes reduced pathogen pressure in introduced ranges facilitates energy allocation toward reproduction and expansion [61].

We acknowledge that our study is limited to only two localities and can be greatly expanded by sampling more populations from different locations. We also note that a key limitation lies in the difficulty in distinguishing between differences in differential gene expression of native and invasive groups due to differences in citric acid exposure, as native populations lack this stressor while invasive ones may face it, potentially skewing our transcriptomic analysis. Nevertheless, we provide sufficient information to suggest that *E. coqui* is under stress and possesses successful genetic toolsets to overcome this stress, enabling it to become an expansive species in both its native and non-native settings. Additionally, we suggest that further sampling of *E. coqui* would greatly aid conservation efforts for other severely endangered *Eleutherodactylus* species.

All our data is publicly available on the NCBI database (BioProject PRJNA953648) and on SourceForge (https://sourceforge.net/projects/coqui-transcriptomes/).

## Supporting information

S1 Fig Draft Mitochondrial Genome of Invasive *E. coqui*, *E. cochranae*, *E. planirostris*, and *P. unistrigatus.* MitoZ and Circos depiction of the mitochondrial genome of Invasive *E. coqui* (A), *E. cochranae* (B), *E. planirostris* (C-D), and *P. unistrigatus* (E). Inner blue circle denotes levels of read mapping from raw Illumina reads.
(DOCX)

S1 Table Differential Expression Analysis Genes of Interest: Native vs Invasive *E. coqui.* (DOCX)

S2 Table Parasite signatures in native *E. coqui* transcriptomes. (XLSX)
