# Supplementary material for: Skin transcriptomics of invasive Coqui frogs: stress responses, parasite signatures, and antimicrobial peptides
Source: PLoS One. 2025 Jul 17;20(7):e0328623. doi: 10.1371/journal.pone.0328623 (PMC12270160; doi:10.1371/journal.pone.0328623)
Supplement: S1 Fig — MitoZ and Circos depiction of the mitochondrial genome of Invasive E. coqui (A), E. cochranae (B), E. planirostris (C-D), and P. unistrigatus (E). Inner blue circle denotes levels of read mapping from raw Illumina reads. (DOCX) [file pone.0328623.s001.docx]

**A**

**
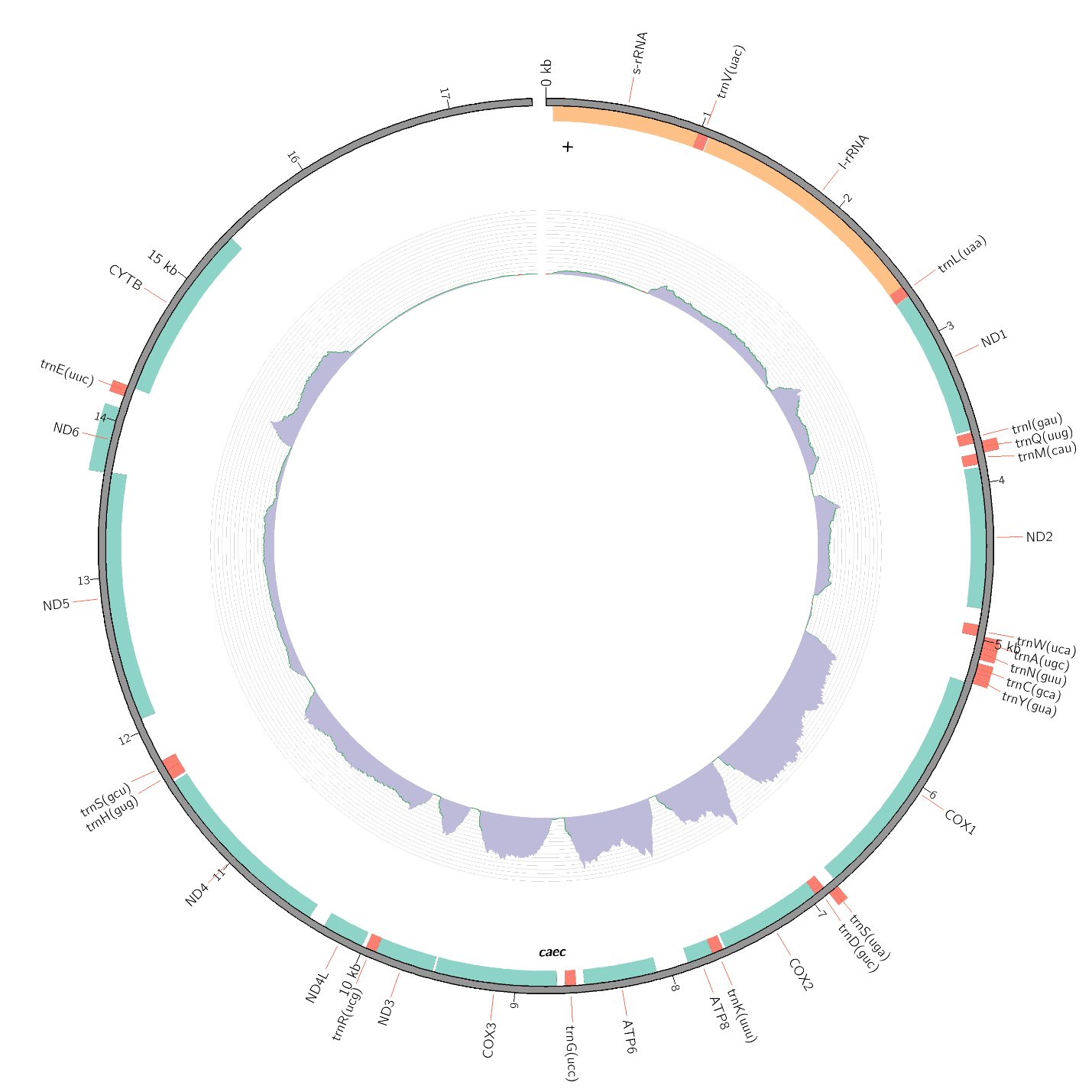
**

**B**

**
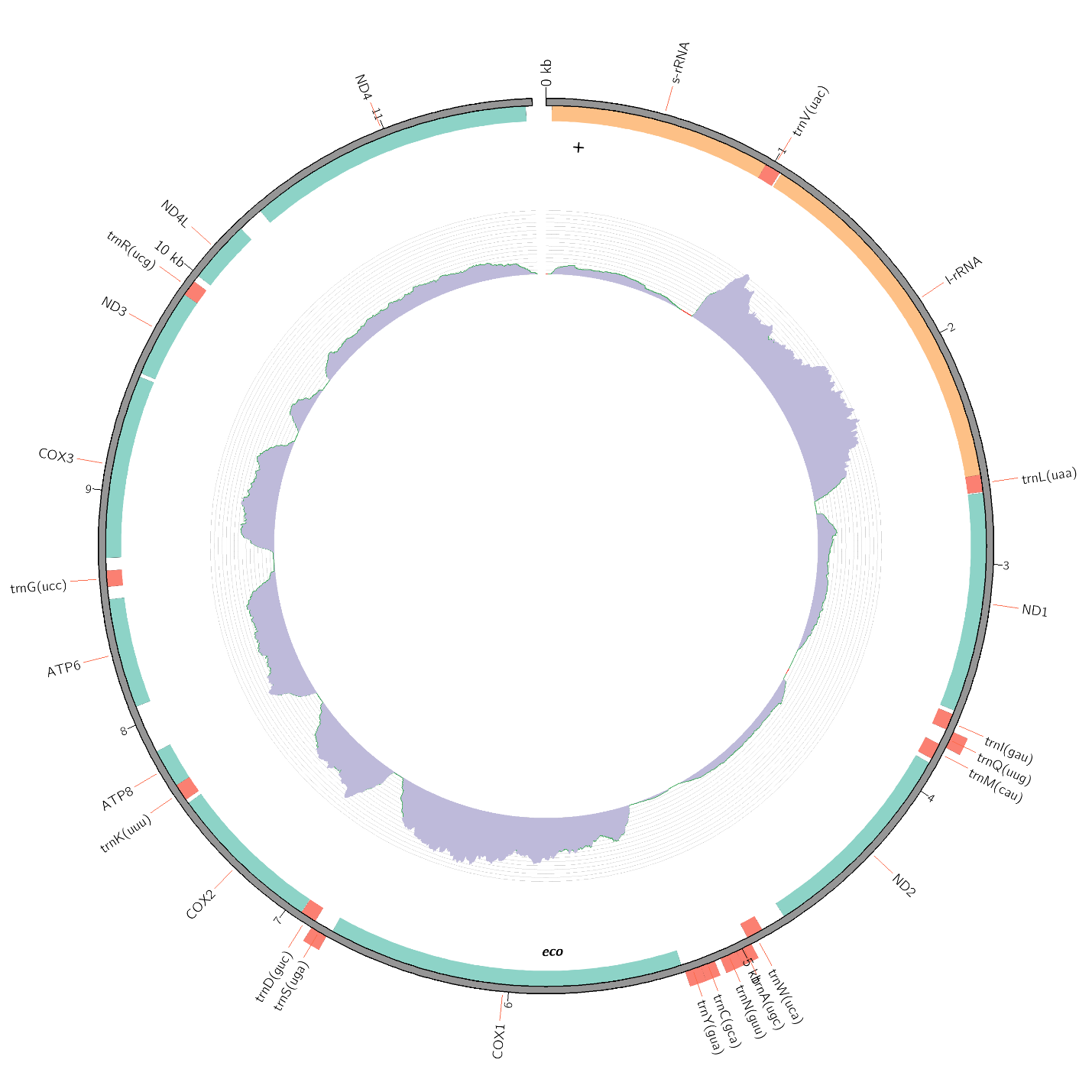
**

**C**

**
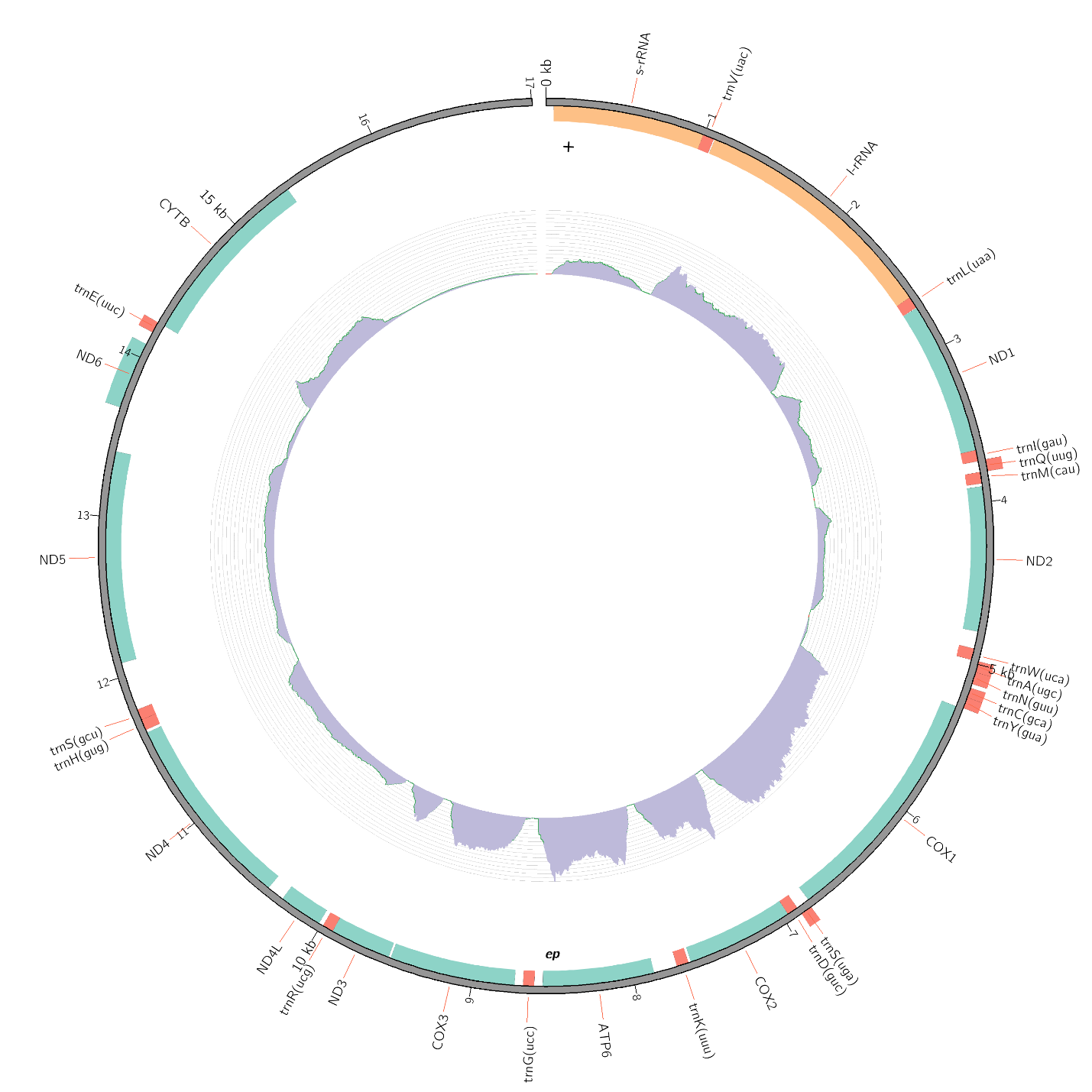
**

**D**

**
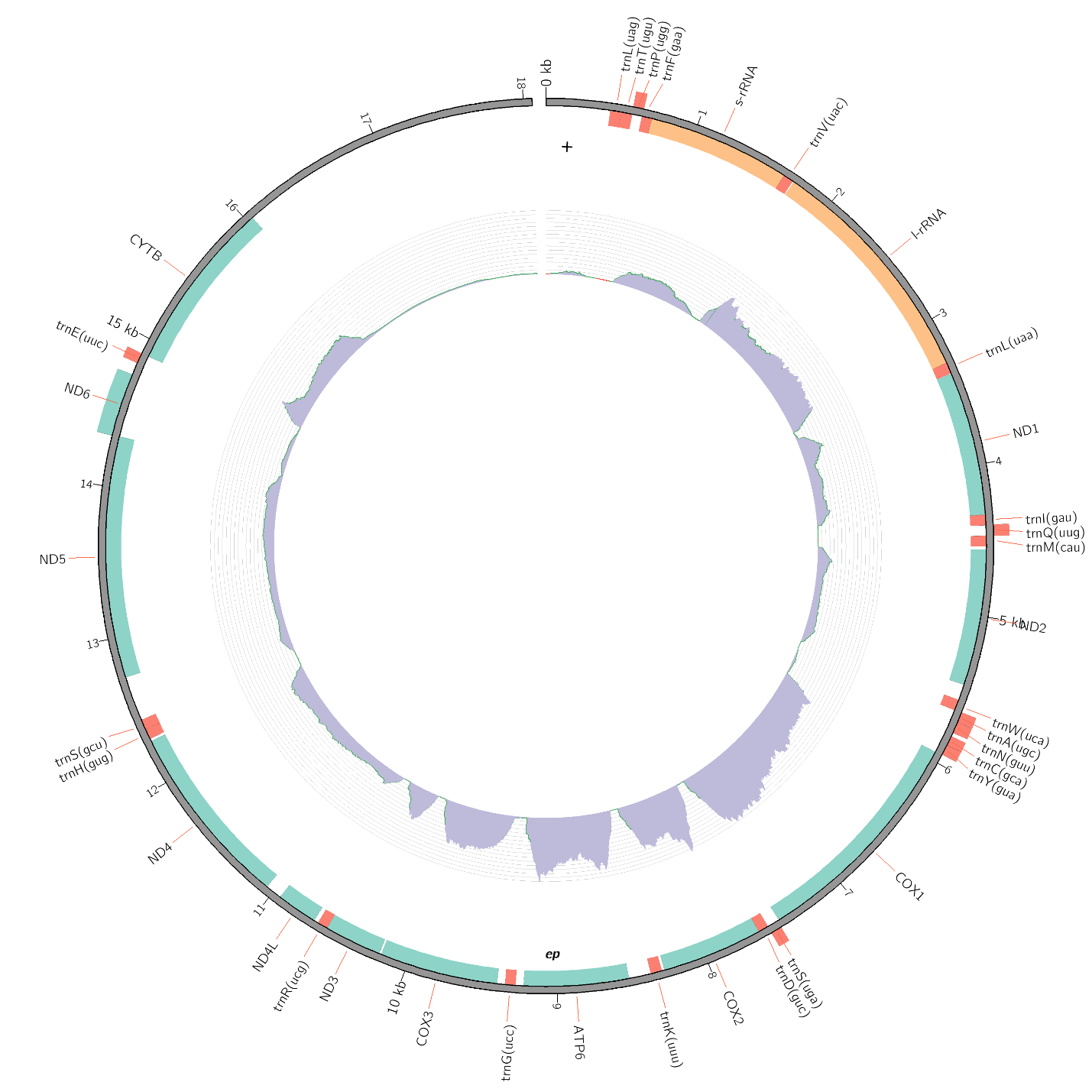
**

**E**

**
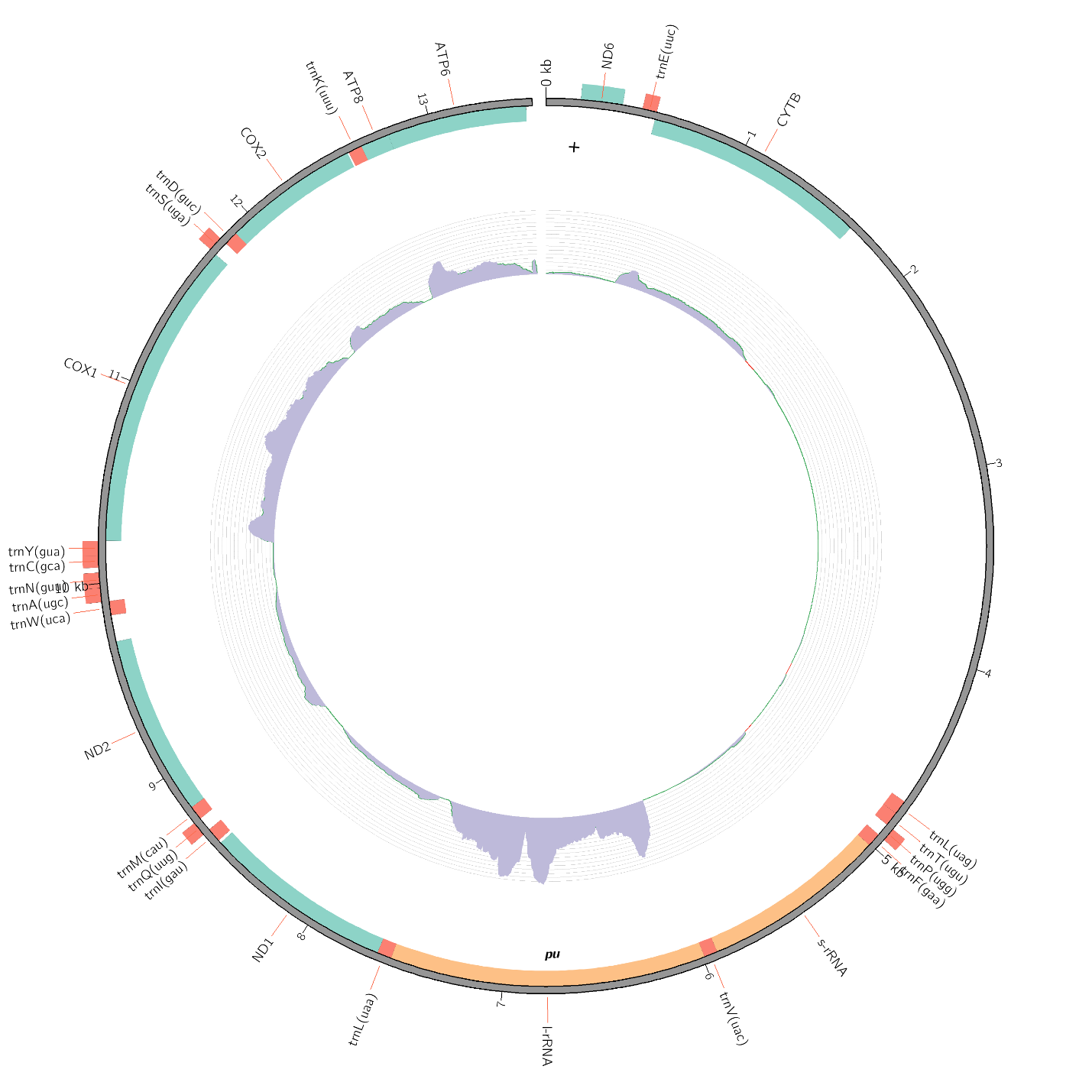
**

**Fig S1 Draft Mitochondrial Genome of Invasive *E. coqui*, *E. cochranae*, *E. planirostris*, and *P. unistrigatus*.**

MitoZ and Circos depiction of the mitochondrial genome of Invasive *E. coqui* (A), *E. cochranae* (B), *E. planirostris* (C-D), and *P. unistrigatus* (E). Inner blue circle denotes levels of read mapping from raw Illumina reads.
