# Supplementary material for: Skin transcriptomics of invasive Coqui frogs: stress responses, parasite signatures, and antimicrobial peptides
Source: PLoS One. 2025 Jul 17;20(7):e0328623. doi: 10.1371/journal.pone.0328623 (PMC12270160; doi:10.1371/journal.pone.0328623)
Supplement: S1 Table — (DOCX) [file pone.0328623.s002.docx]

**Table S1: Differential Expression Analysis Genes of Interest: Native vs Invasive *E. coqui***

| **Full Name** | **Gene Name** | **UniProt ID** | **CA coqui** | **PR coqui** | **foldChange** |
| --- | --- | --- | --- | --- | --- |
| cathelicidin related peptide Oh Cath | CATH | A0A6I8RUJ0 | 0 | 24.01203 | 138.0622 |
| pentraxin 3 | PTX3 | F6VKJ0 | 3.174319 | 25.49348 | 8.081758 |
| reactive oxygen species modulator 1 | ROMO1 | A4QNF3 | 399.9269 | 120.87 | 0.301886 |
| major histocompatibility complex class I related like | MR1-LIKE | A0A803JIS2 | 19.05626 | 0 | 0.010179 |
| mal T cell differentiation protein 2 gene/pseudogene | MAL2 | A0A803JH41 | 87.22814 | 0 | 0.002223 |
| dnaJ heat shock protein family hsp40 member C12 | DNAJC12 | A0A6I8RNZ7 | 13.60621 | 55.83734 | 4.107592 |
| thioredoxin | TRX | A0A803J2A4 | 882.5526 | 330.2608 | 0.374241 |
| MGC76137 protein fragment | MGC76137 | B5DFS7 | 1891.081 | 113.1922 | 0.059886 |
| epiplakin 1 | EPPK1 | A0A803JR42 | 105.1413 | 24.31632 | 0.230981 |
| collagenase 3 | MMP-13 | A0A6I8QBM5 | 24.75311 | 0 | 0.007839 |
| tyrosinase like | TYR-LIKE | A0A6I8QAH5 | 231.9916 | 52.05621 | 0.224716 |
| NADPH dehydrogenase quinone 1 | NQO1 | Q28HD6 | 47.40392 | 9.632187 | 0.20395 |
| ras homolog family member C | RHOC | F7B0F4 | 0 | 98.94468 | 568.4855 |
| ras homolog family member Q | RHOQ | F7CV14 | 42.82706 | 158.8111 | 3.7097 |
| COX7A2 protein | COX7A2 | A4QND9 | 607.7403 | 201.6878 | 0.331722 |
| cytochrome b c1 complex subunit 7 | CYTOCHROME BC1 | A0A6I8Q9X7 | 352.4128 | 142.9203 | 0.405824 |
